# Treatment of Mouse Limb Ischemia with an Integrative Hypoxia-Responsive Vector Expressing the Vascular Endothelial Growth Factor Gene

**DOI:** 10.1371/journal.pone.0033944

**Published:** 2012-03-21

**Authors:** Eduardo Gallatti Yasumura, Roberta Sessa Stilhano, Vívian Yochiko Samoto, Priscila Keiko Matsumoto, Leonardo Pinto de Carvalho, Valderez Bastos Valero Lapchik, Sang Won Han

**Affiliations:** Research Center for Gene Therapy, Department of Biophysics, Universidade Federal de São Paulo, São Paulo, São Paulo, Brazil; University of Colorado Denver, United States of America

## Abstract

Constitutive vascular endothelial growth factor (VEGF) gene expression systems have been extensively used to treat peripheral arterial diseases, but most of the results have not been satisfactory. In this study, we designed a plasmid vector with a hypoxia-responsive element sequence incorporated into it with the phiC31 integrative system (pVHAVI) to allow long-term VEGF gene expression and to be activated under hypoxia. Repeated activations of VEGF gene expression under hypoxia were confirmed in HEK293 and C2C12 cells transfected with pVHAVI. In limb ischemic mice, the local administration of pVHAVI promoted gastrocnemius mass and force recovery and ameliorated limb necrosis much better than the group treated with hypoxia-insensitive vector, even this last group had produced more VEGF in muscle. Histological analyses carried out after four weeks of gene therapy showed increased capillary density and matured vessels, and reduced number of necrotic cells and fibrosis in pVHAVI treated group. By our study, we demonstrate that the presence of high concentration of VEGF in ischemic tissue is not beneficial or is less beneficial than maintaining a lower but sufficient and long-term concentration of VEGF locally.

## Introduction

Peripheral arterial disease (PAD) is characterized by arterial narrowing that reduces oxygen supply to the extremities resulting in severe pain, non-healing ulcers and possible loss of the affected limb. The incidence of PAD is high at about 1000 affected per million individuals, and this incidence increases in individuals over 70 years of age and in diabetics [1]. According to the Transatlantic Inter-Society Consensus, approximately 25% of patients with advanced PAD will suffer amputation because conventional medical and revascularization treatments are not feasible. The prognosis for these patients is bad; after one year, about 25% of them will die, and 20% will still have PAD [1]. Therefore, it is necessary to continue the search for new therapies.

The main cause of PAD is atherosclerosis, which leads to narrowing and malfunctioning of arteries, *i.e.*, ischemia. Consequently, current therapies promote new vessel formation by administering growth factors, which can be provided in protein or gene form or even as cells that produce these factors naturally or after genetic modification [2]–[4]. Among several growth factors used for angiogenic therapy, vascular endothelial growth factor (VEGF) has been the most extensively studied because it is a potent mitogenic factor that also has anti-apoptotic and vessel dilation activities [5], [6]. This factor acts primarily on endothelial cells through the VEGFR1 and VEGFR2 receptors, but it also promotes chemotaxis of smooth muscle cells, monocytes and bone marrow progenitor cells [6]–[8]. In animal studies, VEGF has been shown to improve perfusion and to increase capillary density in ischemic limbs [9]–[15]. Many of these studies have advanced to clinical trials, but most of the results have not been satisfactory.

To date, all clinical trials on limb ischemia treatment have used plasmid or adenoviral vectors designed to express transiently and locally [2]–[4], and muscle has been the favorite target tissue. Transgene expression is transient because the genes are not integrated into the host genome. In clinical trials, these vectors are injected directly into the muscle at high enough doses to express high levels of angiogenic factors due to the characteristics of these vectors. As the angiogenic factor concentration required in loco to induce a therapeutically beneficial amount of angiogenesis is variable depending on the degree of ischemia, angiogenic gene therapy in some tissues can induce hemangioma due to the exaggerated production of growth factors [16], [17], and in other tissues, it cannot ameliorate ischemia due to insufficient production of the angiogenic factor. Thus, the ideal vector system should have a mechanism to allow for the long-term production of angiogenic factors according to the local degree of ischemia.

To obtain a vector that is responsive to ischemia and is capable of long-term VEGF expression, we designed an integrative plasmid vector based on the ΦC31 integrase system that contains an HRE (hypoxia-responsive element) sequence. The HRE consensus sequence, isolated from the 3′ end of the erythropoietin (Epo) gene, is present in many genes involved in erythropoiesis, angiogenesis and glycolysis [18] that are activated during hypoxia. Cells and tissues exposed to hypoxia trigger an adaptive response driven by hypoxia induced factor 1 (HIF-1), which binds to the HRE sequence located in the enhancer regions of these genes. It has been demonstrated that vectors constructed with an HRE sequence provide enhanced transgene expression under hypoxic conditions through HIF-1 binding to the HRE [18], [19]. In addition, to allow for integration of the vector, the attB sequence from phage ΦC31 was included. The integrase from ΦC31 recognizes plasmids with the attB sequence and inserts them into genomic regions containing pseudo-attP sequences [20], which are present in mammalian genomes. Because the integration process is unidirectional, once the plasmid is inserted into the genome, it is maintained stably [21]. In our study, we found that limb ischemic mice treated with our integrative vector that is responsive to variations in oxygen concentration had a better therapeutic response than mice treated with other delivery vectors.

## Materials and Methods

### Vector construction

**Figure 1:**
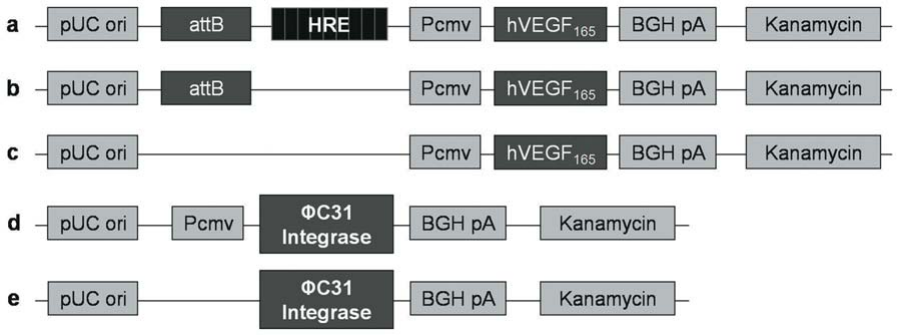
Schematic vector diagrams. (A) pVAX-HRE-attB-hVEGF165; (B) pVAX-attB-hVEGF165; (C) pVAX-hVEGF165; (D) uP-INT; (E) p-INT. pUC ori: replication origin. Pcmv: human cytomegalovirus promoter. BGHpA: polyadenylation signal of the bovine growth hormone. Kanamycin: kanamycin resistance gene.

The commercially available pVAX vector (Invitrogen, Carlsbad, EUA) was cut with HincII enzyme, and a cassette containing 9 copies of the HRE sequence (CCGGGTAGCTGGCGTACGTGCTGCAG) from the AAV-H9-lacZ vector (kindly provided by Dr Hua Su, The Cardiovascular Research Institute, University of California, USA [22], which was obtained by digesting with EcoRI and BglII and treating with Klenow polymerase, was ligated to make pVAX-HRE. This vector was digested with NruI and was ligated with the attB sequence from the pTA-attB vector (kindly provided by Dr Michele P. Calos, Genetics department of Stanford University, USA [20]), which was previously digested with BamHI and EcoRV and treated with Klenow polymerase, to make pVAX-HRE-attB. Finally, to insert the human VEGF165 sequence into pVAX-HRE-attB, both it and the uP-VEGF vector [23], which expresses human VEGF165, were digested with HindIII and ApaI and ligated together to make pVAX-HRE-attB-VEGF. The integrase expression vector uP-INT was constructed by inserting the ΦC31 integrase gene into the uP vector. The p-INT vector has a construction similar to uP-INT, but it does not have a CMV promoter; therefore, it does not express integrase (Fig. 1).

### Cell culture under normoxia and hypoxia

The human embryonic kidney cell line HEK293T [24] was maintained in DMEM supplemented with 10% fetal bovine serum (DMEM+). For transfection, in a 6-well plate 1×10^5^ HEK 293 cells were seeded. Twenty-four hours later, 2.5 µg of the donor plasmid containing hVEGF_165_ and 2.5 µg of pVAX or p-INT or uP-INT plasmids were mixed for transfection by the calcium-phosphate co-precipitation method. After 24 h, the medium was replaced with a fresh one, and at the indicated time, the supernatant was collected to determine VEGF levels by ELISA.

To establish hypoxic condition in vitro, the cells were cultured with DMEM+ containing 100 µM cobalt chloride [25]. To test the toxicity of the cobalt, HEK293 cells were cultured with 0, 200 or 400 µM cobalt chloride, and cell viability was assessed using the Trypan blue method.

The mouse myoblast cell line (C2C12) was also maintained in DMEM+, but for transfection the Amaxa NHDF Nucleofector kit and Nucleofector Amaxa (Lonza, Basel, Switzerland) were used following the provided protocol.

### Ischemic hind limb model and muscular transfection

All procedures involving animals were approved by the Research Ethics Committee of the Federal University of São Paulo, Brazil (Approval number: CEP 0729/08). Initially, 10–12 week-old Balb/c male mice were anesthetized with an intraperitoneal injection of ketamine (40 mg/kg) and xylazine (20 mg/kg). Hind limb ischemia was induced surgically as previously described [26], [27]. Briefly, the femoral artery was excised from its origin at the external iliac artery branch to its bifurcation into the saphenous and popliteal arteries without damaging the femoralis vein or nerves. Branches including the circumflex artery were also obstructed completely to avoid retrograde flow. Gene therapy was performed by injecting 50 µg of each vector in 100 µl of phosphate-buffered saline (PBS) into the middle of the quadriceps muscle soon after ischemic surgery. After plasmid injection, 3 electric pulses of 80 V/cm and 20 ms in duration were applied using needle electrodes (Electroporator T820; BTX Genetronics, San Diego, USA). After gene therapy, the animals were kept under analgesia with daily peritoneal injections of 5 mg/kg carprofen. In our study, the following groups were included with 7–10 animals per group: normal, ischemic and ischemic treated with vector.

### Visual assessment of necrosis

Limb ischemia was visually evaluated 30 days after treatment. The following four grades were used to measure the degree of limb necrosis: grade 0, absence of necrosis; grade 1, necrosis limited to the toes; grade 2, necrosis extending to the dorsum pedis; grade 3, necrosis extending to the crus [26].

### Muscle force determination

Isometric gastrocnemius muscle contraction was performed 30 days after treatment based on a previous study [28]. The animals were anesthetized and maintained at 37°C on a temperature-controlled plate. The leg and knee were fixed on the plate with the gastrocnemius muscle exposed. The Achilles tendon was cut and attached to a wire connected to a force transducer (MLT 1030/D - ADInstruments, Bella Vista NSW, Australia). The lateral gastrocnemius and soleus muscles were removed; the vascular and nerve systems were kept intact. Medial gastrocnemius muscles were left free from the surrounding tissue to minimize effects from the contraction of other muscles. The sciatic nerve was isolated and placed in contact with a bipolar silver electrode for electro-stimulation. One stimulus of 5 volts at a frequency of 60 Hz was applied for 10 ms with a 1 minute interval between stimulations. The isometric contractions were measured as the highest force stretching the wire. All collected data were recorded and analyzed using the PowerLab 8/30 system and LabChart Pro software (ADInstruments, Bella Vista NSW, Australia). After force determination, the animals were subjected to euthanasia, and their medial gastrocnemius muscles were then removed for mass measurement and histological analysis.

### Determination of hVEGF_165_ by ELISA

Supernatant from cultured cells was collected periodically to determine the concentration of hVEGF_165_ using a DuoSet ELISA kit (R&D Systems Inc., Minneapolis, USA) following the manufacturer's instructions. To determine hVEGF_165_ levels in muscle, the quadriceps muscle was removed after muscle force measurement. The middle part of the gastrocnemius muscle was mechanically homogenized using lysis buffer (25 mM Tris-HCl, pH 7.4, 50 mM NaCl, 0.5% Na-deoxycholate, 2% NP-40, 0.2% sodium dodecyl sulfate, 1 mM phenylmethylsulphonyl fluoride). The homogenized tissue was centrifuged at 4500× g for 10 min at 4°C, and the supernatant was recovered. Total protein and hVEGF_165_ concentrations were determined using the Bio-Rad Protein Assay (BioRad, California, USA) and a DuoSet ELISA Kit, respectively.

### Gene expression analysis by quantitative reverse transcription polymerase chain reaction (qRT-PCR)

Total RNA from C2C12 or HEK293 cells was extracted with Trizol reagent (Invitrogen, Carlsbad, CA, USA) and treated with DNAse I (Invitrogen). cDNA was synthetized using High Capacity cDNA Reverse Transcription kit (Invitrogen) and qRT-PCR was conducted using QuantiFast SYBR Green RT-PCR Kit (Qiagen, Hilden, Germany) in the Rotor Gene-Q (Qiagen). The following primers were used to quantify mouse HIF1- α (m HIF1- α), human HIF1- α (h HIF1- α) and human VEGF (hVEGF): mHIF1-α_F (gca gca gga att gga acat t), mHIF1-α_R (gcat gct aaa tcg gag ggta), hHIF1-α_F (caa gaa cct act gct aat gc), hHIF1-α_R (tta tgt atg tgg gta gga gat g), hVEGF_F (ttc tgc tgt ctt ggg tgc att gg), hVEGF_R (gaa gat gtc cac cag ggt ctc g). Relative gene expression was calculated by 2^−ΔCT^. The changes in mRNA expression were expressed as fold-changes relative to control, which was the RNA from C2C12 or HEK293 cells without cobalt and transfection. As normalizer of qRT-PCR, the ribosomal gene hRPS29 was used with primers hRPS29_F (gag cca ccc gcg aaa at) and hRPS29_R (ccg tgc cgg ttt gaa cag) for HEK 293 and the murine β-actin gene with primers mβ-actin_F (gct cct cct gac cgc aag) and mβ-actin_R (cat ctg ctg gaa ggt gga ca) for C2C12. Each reaction was carried out in duplicate and all experiments were carried more than three times. Values were expressed in the mean ± standard error of the mean. One way ANOVA with Bonferronis's multiple comparison was used to statistical analysis.

### Vector integration analysis by PCR

Genomic DNA was extracted from quadriceps muscles or from transfected HEK293 cells using the QIAMP DNA Mini Kit (QIAGEN Inc., Valencia, USA). To check for vector integration into the host genome, a PCR reaction was carried out using a pair of primers, CMV reverse 5′-TCATTATTGACGTCAA TGGGC-3′ and attB sense 5′-CTCCACCTCACCCATCT-3′, at a final concentration of 0.5 µM. The PCR reaction was performed by programming a thermocycler to 94°C for 1 min and 35 cycles of 94°C/1 min, 52°C/1.5 min and 72°C/1 min. After the final cycle, the reaction continued for 7 min at 72°C, and the tubes were then maintained at 4°C. As an internal control for the PCR, the glyceraldehyde-3-phosphate dehydrogenase (GAPDH) housekeeping gene was used with GAPDH sense (5′-ACCACAGTCCATGCCATCAC-3′) and GAPDH antisense (5′-TCCACCACCCTGTTGCTGTA-3′) primers. The PCR products were analyzed by 1% agarose gel electrophoresis with ethidium bromide staining.

### Histological analysis

Muscle samples were fixed in 10% formaldehyde, dehydrated and embedded in paraffin. Four micrometer sections were obtained and stained with hematoxylin-eosin (HE) and picrosirius. The extent of necrosis, muscle regeneration and fibrosis were analyzed from 20 fields and quantified using Image Pro Plus software (Media Cybernetics, Inc., Bethesda, USA). Other sections were collected in poly L-lysine coated slides and submitted for immunohistochemistry with biotinylated Griffonia (bandeiraea) simplicifolia lectin I (1∶100) (Vector Laboratories, Peterborough, UK) or anti-alpha smooth muscle actin antibody (1∶50) (Vector Laboratories, Peterborough, UK) followed by incubation with streptavidin conjugated peroxidase (Sigma-Aldrich, Saint Louis, USA) and detection with diaminobenzidine chromogen. Vessel and capillary densities were quantified from 20 random fields per slide. The results were expressed as the average number per square millimeter.

### Statistical analysis

The results were expressed as mean ± standard error of the mean. Analysis of variance (ANOVA) was performed using a Bonferroni correction for multiple comparisons or a Mann-Whitney test for a comparison of two groups. Visual assessment of necrosis was analyzed by the one way ANOVA with Tukey's multiple comparison tests. Only P<0.05 was considered significant.

## Results

### In vitro evaluation of VEGF gene expression

**Figure 2:**
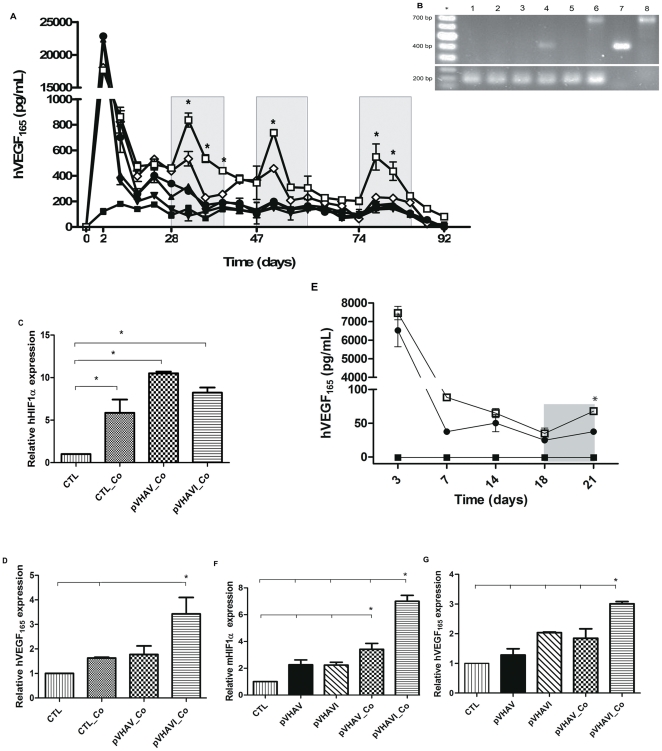
Assessment of VEGF expression vectors. (A) hVEGF_165_ production under hypoxia and normoxia in HEK293 cells. On days 28, 47 and 74, CoCl_2_ was added to a final concentration of 100 µM, and the medium was replaced every 3 days with fresh medium containing CoCl_2_ for 9 days (gray bars). -▪- no transfection; -▴- pVV; -▾- pVAV; -◊- pVAVI; -•- pVHAV; -□- pVHAVI. * p<0.001, pVHAVI in comparison to all groups. (B) Genomic DNA PCR after 4 weeks of transfection. 1: no transfection; 2: pVV; 3: pVAV; 4: pVAVI; 5: pVHAV; 6: pVHAVI; 7: pVAX-attB-hVEGF_165_ (432 bp); 8: pVAX-HRE-attB-hVEGF_165_ (725 bp). The 200 bp band is from GAPDH. * 100 bp ladder. pVV: pVAX-hVEGF_165_ + pVAX; pVAV: pVAX-attB-hVEGF_165_ + p-INT; pVAVI: pVAX-attB-hVEGF_165_ + uP-INT; pVHAV: pVAX-HRE-attB-hVEGF_165_ + p-INT pVHAVI: pVAX-HRE-attB-hVEGF_165_ + uP-INT (C) HIF1α and (D) hVEGF_165_ expression in HEK293 cells. On day 15 the HEK293 cells from the experiment (A) were collected for analysis by qRT-PCR. CTL: control; Co: CoCl_2_ ; * p<0.05. (E) hVEGF_165_ production under hypoxia and normoxia in C2C12 cells. On day 18 CoCl_2_ was added to a final concentration of 100 µM and 3 days later the medium was collected for VEGF quantification by ELISA. -▪- no transfection; -•- pVHAV; -□- pVHAVI. * p<0.05. (F) HIF1α and (G) hVEGF_165_ expression in C2C12 cells. On day 15 the C2C12 cells from the experiment (C) were collected for analysis by qRT-PCR. CTL: control; Co: CoCl_2_ ; * p<0.05 of pVHAVI_Co group versus all other groups.

To assess the functionality of the constructed vectors (Fig. 1), HEK293T cells were transfected with these vectors, and VEGF gene expression was monitored for more than 90 days (Fig. 2). The calcium co-precipitation method was used to transfect HEK293T cells because this method achieves more than 90% transfection efficiency in this cell line (data not shown). To induce hypoxia, CoCl_2_ was added to the medium at a final concentration of 100 µM, which is a concentration commonly used to mimic hypoxia [24]. HEK293T cells incubated with 100 µM CoCl_2_ for nine days retained about 90% cell viability (data not shown).

Under normoxia conditions, all groups showed a peak of VEGF gene expression at 48 hours post-transfection, which is a well-known transient gene expression pattern, but by the 14^th^ day, VEGF expression in all groups had returned to basal levels (Fig. 2). Cells transfected with the integrative vectors pVAVI and pVHAVI still maintained about 500 pg/ml VEGF expression, whereas those transfected with non-integrative vectors expressed about half of this value. On the 28^th^ day, CoCl_2_ was added to the culture medium and maintained for 9 days. Three days after CoCl_2_ addition, VEGF production was found to be enhanced only in the pVHAVI transfected cells; it reached about 900 pg/ml but returned to basal levels later. The pVAVI group did not show any increase in VEGF levels during the first 3 days, and these levels diminished even more in subsequent days. Upon removing CoCl_2_, VEGF gene expression levels returned to their initial status.

Activation of VEGF gene expression by CoCl_2_ was repeated on the 47^th^ and 74^th^ days and resulted in a very similar pattern of gene expression. After the third stimulation, the experiment was stopped because most of the cells became unviable. Long-term cell culturing with CoCl_2_ seems to affect cell viability. These results clearly demonstrate that the vectors functioned correctly, particularly the pVHAVI system, which is the only system that is expected to be hypoxia-responsive.

To verify genomic integration of the pVHAVI and pVAVI vectors, genomic DNA was extracted 30 days after transfection, and a known region of the vectors was amplified by PCR. Fig. 2B shows the amplification of 735 bp and 432 bp products, which correspond to the pVAX-HRE-attB-hVEGF_165_ and pVAX-attB-hVEGF_165_ vectors, respectively. DNA from the other vectors was not amplified by PCR.

To demonstrate the activation of HIF1α by CoCl_2_, the expression of HIF1α and hVEGF genes was evaluated by qRT-PCR at the 15^th^ day. Irrespective of the presence or not of vectors, the presence of CoCl_2_ in the medium elevated HIF1α gene expression 7 to 11 folds in relation to cells without CoCl_2_ (Fig. 2C); however, only those cells transfected with pVHAVI had elevated VEGF gene expression in the presence of CoCl_2_ (Fig. 2D), indicating the correct functioning of the pVHAVI system.

To strengthen these findings, we tested pVHAVI system in the murine myoblast cell line C2C12, which is the main cell type present in skeletal muscles. To transfect this cell line we chose nucleofection method, because we had very low level of transfection with calcium phosphate co-precipitation method (not shown). Even using nucleofection the VEGF gene expression levels were lower than HEK293 cells, but the profile of gene expression over 21 days was very similar (Fig. 2E). At the 3^rd^ day after transfection, VEGF gene expression reached about 7,000 pg/ml by both pVHAVI and pVHAV systems, but in a week it dropped to basal level and, at 14^th^ day, there was no significant difference of gene expression between these two systems.

CoCl_2_ was added to cell culture media at the 18^th^ day, but only those cells transfected with pVHAVI had increased hVEGF gene expression, meanwhile the cells transfected with pVHAV or non-transfected ones did not have any significant alterations. Activation of HIF1α by CoCl_2_ was also seen in C2C12 cells (Fig. 2F), which was about 7 and 4 folds higher than control group by pVHAVI and pVHAV systems, respectively, but only pVHAVI transfected cells had increased hVEGF gene expression (Fig. 2G).

### Visual, functional and molecular analyses of muscles after gene therapy

**Figure 3:**
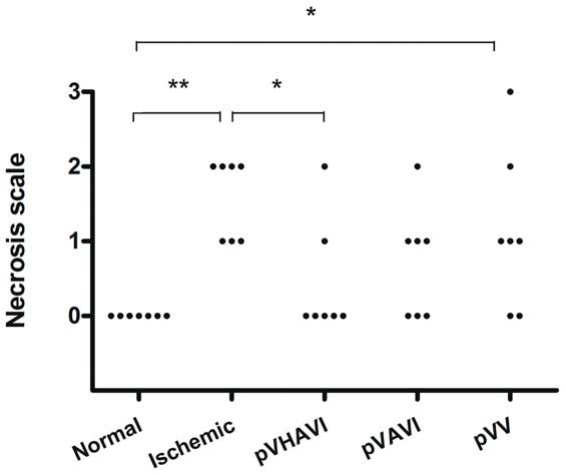
Visual assessment of ischemic limbs after gene therapy. Four weeks after gene therapy, limbs were evaluated according to the following necrosis scale: grade 0: absence of necrosis; grade I: necrosis limited to the toes; grade II: necrosis extending to the dorsum pedis, grade III: necrosis extending to the crus. * *P*<0.05; ** *P*<0.005.

Visual assessment is an easy method to carry out and provides consistent and relevant information. To make this type of assessment quantitative, the degree of necrosis was scored as described in the “Materials and Methods” section. All animals in the ischemic group without gene therapy presented grade 1 or 2 necrosis. Two animals from the pVV group presented no necrosis, demonstrating the therapeutic effect of the treatment, but the rest of group still showed some degree of necrosis. The pVAVI-treated animals had a better outcome than the pVV group because about half of them had no necrosis. However, the best result was obtained with pVHAVI treatment, which resulted in no visual necrosis in any animals in the group (Fig. 3).

**Figure 4:**
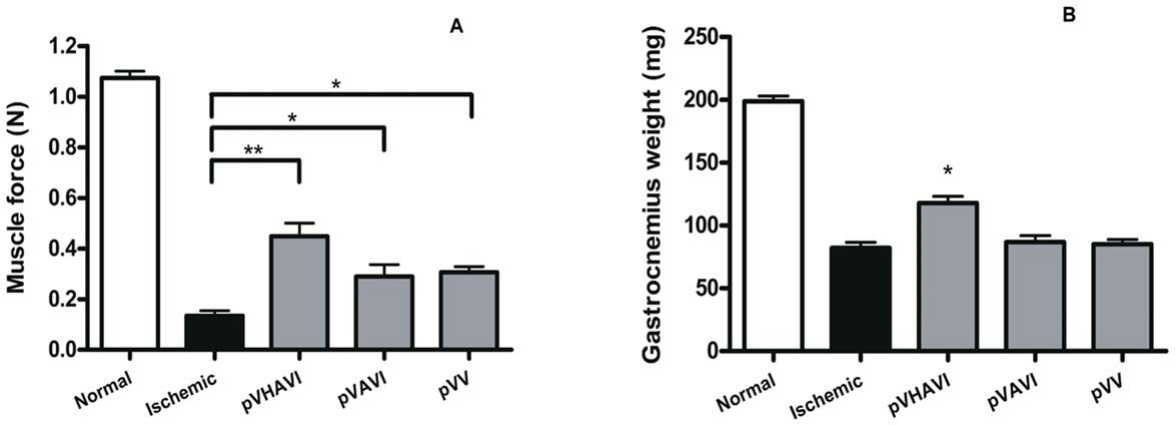
Determination of the mass and force of the gastrocnemius muscle. Muscle force (A) and mass (B) were measured 4 weeks after gene therapy. For each group, seven mice were used. The sham-operated and non-ischemic groups showed no difference in their results and are denoted here as normal. (A) * *P*<0.05; ** *P*<0.005. (B) * P<0.05, pVHAVI in comparison to each group.

To assess muscle functioning, the gastrocnemius muscle force was determined after 4 weeks of gene therapy. Ischemic limbs showed a drastically reduced force from 1.04 N to 0.12 N, but pVHAVI-treated animals exhibited a force of about 0.46 N, which is equivalent to a 400% improvement. pVV- and pVAVI-treated animals reached an intermediate score of 0.3 N (Fig. 4A). The weight of the gastrocnemius muscle varied among the groups in a pattern similar to that of the muscle force variation. In the pVHAVI-treated animals, this weight was about 50% that of the non-ischemic animals, whereas in the other groups, it was only about 36% (Fig. 4B). It is important to note that the untreated ischemic animals had a similar muscle weight compared to those treated with pVV or pVAVI, but in terms of muscle force, the untreated ischemic group had a much lower value. Muscle force depends on the number and volume of correctly functioning muscle fibers, whereas muscle mass encompasses the mass of all tissues including non-contractile fibrotic tissues, the amount of which varies with disease evolution. Therefore, a minor variation between muscle mass and force after 4 weeks of ischemia is expected.

**Figure 5:**
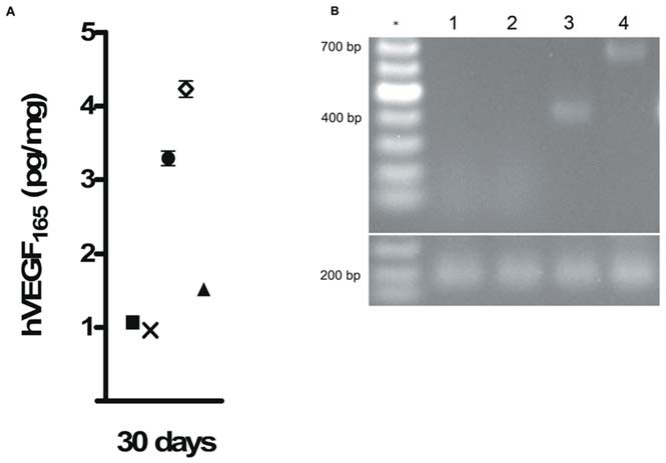
Assessment of VEGF expression vectors after gene therapy. (A) Concentration of hVEGF_165_ in the quadriceps muscle: -**x**- Ischemic; -▪- Normal; -▴- pVV; -•- pVHAVI; - **◊** - pVAVI. (B) Genomic DNA PCR after four weeks of gene therapy. 1: normal; 2: pVV; 3: pVAVI (432 bp); 4: pVHAVI (725 bp). The 200 bp band is from GAPDH. * 100 bp ladder.

The pVAVI and pVHAVI vectors are integrative systems, and consequently, gene delivery using these vectors is expected to result in long-term gene expression. To validate the correct functioning of these vectors, VEGF gene expression was evaluated in both serum and muscle tissue. In serum, VEGF was not detected at any time in any group. Muscle extracts obtained after 30 days from the pVHAVI and pVAVI groups had about 3.3 and 4.2 pg/mg of VEGF, respectively, and the ischemic and pVV-treated groups had less than 1.5 pg/mg (Fig. 5A). As 1.5 pg/mg is at the limit of VEGF detection by ELISA, we considered this to be a null value.

To check for vector integration in host cells, PCR was performed using genomic DNA obtained from muscles as a template. DNA bands of 735 bp and 432 bp were detected in the pVHAVI and pVAVI groups, respectively, and these correspond to the pVAX-HRE-attB-hVEGF_165_ and pVAX-attB-hVEGF_165_ vectors, respectively (Fig. 5B). Other groups showed no amplification, indicating that no vector integration had occurred after 30 days.

### Histological analyses

**Figure 6:**
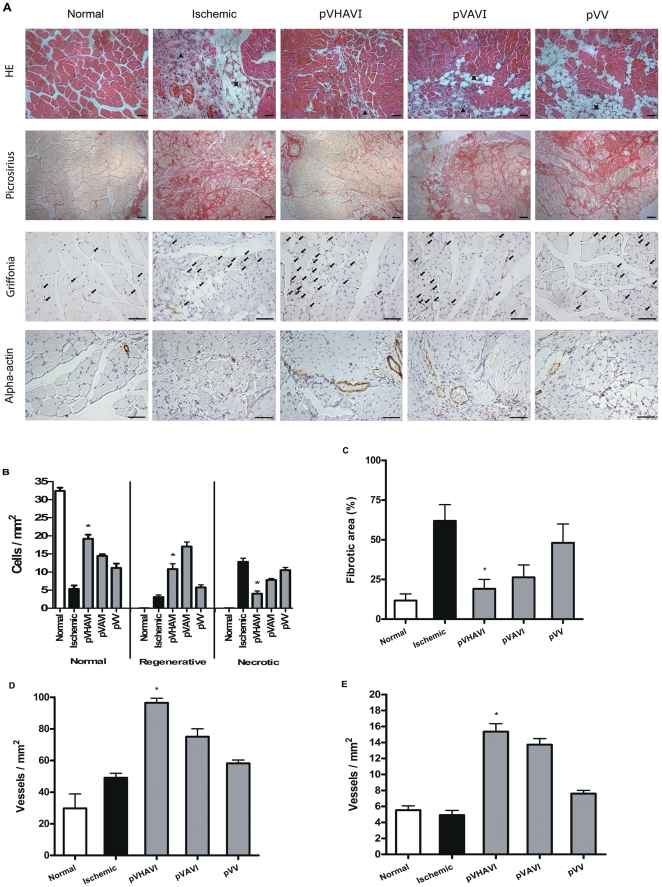
Morphometric analysis of limb muscles. The gastrocnemius muscle was collected from mice after four weeks of gene therapy. Tissue samples were stained with HE (A) and used to quantify necrotic, regenerative and normal areas (B). The sham-operated and non-ischemic groups showed no difference in their results and are denoted here as normal. Fibrotic area, capillary density and mature vessel density were determined after staining with Picrosirius (C), Griffonia (D) and alpha-actin antibody (E), respectively. Bar = 50 µm. * p<0.05. ▴, Infiltrated mononuclear cells; X, adipocytes; →, capillary; In the figure B, pVHAVI was different statistically in comparison to all groups.

Gastrocnemius muscles were stained with HE, and the numbers of necrotic, normal and regenerative cells were quantified (Fig. 6A and 6B). The ischemic group showed about 15 cells/mm^2^ of necrotic cells, and this was reduced to 5 cells/mm^2^ in the pVHAVI-treated group. Moreover, the normal cell count increased from 5 cells/mm^2^ in the untreated ischemic group to almost 20 cells/mm^2^ in the pVHAVI-treated group. The groups treated with the other vectors showed intermediate values. In terms of regenerative cell count, the pVHAVI group (12 cells/mm^2^) had a smaller count than the pVAVI group (18 cells/mm^2^) but a much higher count than the ischemic group (4 cells/mm^2^).

A similar order of fibrotic area was seen after staining with Picrossirus (Fig. 6C): ischemic>pVV>pVAVI>pVHAVI>normal. These findings corroborate the previously obtained results demonstrating that the therapeutic effect of pVHAVI is better than that of either the non-integrative pVV system or the integrative and constitutive pVAVI system.

To evaluate angiogenic activity promoted by VEGF, vessels were stained with lectin Griffonia, which recognizes endothelial vessels and macrophages. This staining system was chosen over staining with an anti-CD31 antibody to allow both vessels and infiltrated macrophages to be visualized in the same section. Macrophages and endothelial cells can be differentiated easily by optical microscopy using high magnification. In addition, the anti-alpha-actin antibody was used to localize mature and larger vessels. The number of vessels stained with the two systems was similar (Fig. 6D and 6E). The pVHAVI-treated group had the highest number of vessels followed by the pVAVI and the pVV groups. Even though the difference between the pVHAVI and pVAVI groups was not statistically significant, the pVHAVI treatment tended to be superior.

## Discussion

Since the first clinical trial of gene therapy for the treatment of ischemic limbs (which used a plasmid vector expressing the VEGF gene) by Jeffrey Isner in 1996 [29], more than 150 gene therapy trials have been initiated, and about half of them are still opened (http://www.wiley.co.uk/genetherapy/clinical/). The VEGF gene has been widely used in preclinical and clinical assays to treat ischemic diseases because it is a pleiotropic factor involved in many cellular activities, most of which are closely related to angiogenesis. In most of the animal studies, vectors expressing VEGF have been injected directly into the ischemic tissue, and this was found to improve blood flux and transiently augment vessel density [2]. However, we and other groups have demonstrated that long periods of high expression of this gene in ischemic tissue can lead to deleterious effects, such as a decrease in capillary density, the loss of muscle mass and force and the augmentation of limb necrosis [27], [30], [31]. It is likely that the high concentration of VEGF provided by the ischemic tissues together with the transfected cells promotes fast endothelial cell proliferation and vessel formation, which are not followed by adequate vessel maturation. These premature vessels in muscles can be easily disassembled by muscular movements, potentially leading to edema and cell death. Using VEGF together with either the stem cell mobilizing factor G-CSF (granulocyte colony stimulating factor) or the arteriogenic and vasculogenic factor GM-CSF (granulocyte macrophage-colony stimulating factor) to treat mouse ischemic limbs has resulted in much better outcomes than treatment with VEGF alone [26], [27]. Our interpretation of these results is that, to make a stable and functional vessel, it is necessary to recruit more growth factors and cells in an adequate time frame and at adequate concentrations, as happens physiologically.

In angiogenic gene therapy assays, plasmid and adenoviral vectors are primarily used because they provide efficient gene transfer to muscles in vivo and express transgenes transiently. Most (if not all) of these vectors are designed to express transgenes highly and continuously using strong constitutive promoters, with the expectation that the secreted angiogenic factors will be spread around the whole ischemic area. However, most angiogenic factors like VEGF contain a heparin-binding domain in their structure [32]–[34], which causes these factors to be retained around the production area. Consequently, an area with cells transfected with VEGF produces this factor continuously irrespective of the local production due to ischemia, making the local concentration higher than necessary after a certain period of time. Such a condition usually leads to the formation of unstable, immature and hypofunctional vessels [16], [35]. The best way of expressing VEGF for therapy is ideally by using vectors that respond to the requirements of the local tissue, *i.e.*, vectors that are regulated by the local degree of ischemia, as normal cells are.

Physiological VEGF gene expression is modulated by the local oxygen concentration. In ischemic tissue, the oxygen supply is limited, and oxygen distribution occurs mostly by passive diffusion from arteries to tissues [36]. This is one of the reasons that limbs distant from the obstructed artery are more affected than proximal limbs. Therefore, it is expected that the degree of ischemia, or the degree of oxygenation, will be variable in different parts of ischemic tissue. In this manner, VEGF production should correlate with either the degree of ischemia or the oxygen concentration. In humans, to overcome the ischemic condition, the ischemic tissues naturally express angiogenic factors based on the local oxygen concentration, such as VEGF, which is monitored by HIF-1 [37]. HIF-1α translocates to the nucleus during hypoxia, where it associates with HIF-1β to make a dimer, which in turn activates genes containing HRE (hypoxia responsive element) sequences. Genes involved in survival under hypoxic conditions, such as those that regulate angiogenesis, vessel dilation, erythropoiesis and glycolysis, are regulated by the binding of HIF to the HRE [19], [37].

To make a VEGF-expressing vector responsive to hypoxia, we used nine repeats of the HRE sequence, which is responsive to HIF. A vector with nine repeats was used because it functioned better than vectors with fewer repeats [38]. In addition, with the goal of making an integrative vector capable of providing long-term VEGF expression, the phiC31 integrase system was used. This system allows integration of the vector in one direction, *i.e*., once the vector is integrated into the host genome it cannot be removed enzymatically [20], [21]. Using this system, our expectation was that one treatment of an ischemic limb with the vector (pVHAVI) would be sufficient for a long period, and VEGF gene expression would be regulated by the vector itself.

To demonstrate that the pVHAVI system was functioning correctly, these vectors were tested *in vitro* using HEK293T cells and myoblast cell line C2C12 and *in vivo* using a mouse ischemic limb model. In our *in vitro* study, HEK293T cells were transfected with several vectors (Fig. 1), and VEGF gene expression was followed for more than 90 days. The pVHAVI system was the only one that responded to hypoxia, which was induced by CoCl_2_ to stabilize HIF1α from degradation. It is important to note that hypoxia was induced at three different times and the cells modified with pVHAVI responded precisely to the hypoxic signal each time. A very similar result was also seen in C2C12 cell line. These results demonstrate clearly that VEGF expression can be activated at any moment by hypoxia, as we predicted.

Correct functioning of the pVHAVI system, as indicated by the induction of VEGF gene expression by hypoxia, was observed for a month. In this study, we opted to use the limb ischemia model rather than administration of CoCl_2_ because the first model is much more similar to human ischemic disease, and it is a well-established animal model [26], [27], [39]. Additionally, we have not found a method for using CoCl_2_ to induce ischemia in vivo or any other method that can induce limb ischemia repeatedly without significantly affecting the animal's physiology. As a result, we could not induce hypoxia repeatedly in animals to evaluate the functioning of the pVHAVI system over a long period as we could for the in vitro model. Consequently, we chose to evaluate the system indirectly after gene therapy by examining therapeutic parameters such as alterations in muscle mass, force and histology and by visually assessing muscle necrosis. For in vivo gene transference, electroporation was used because in our previous studies, we demonstrated that this method is reproducible and results in high levels of transfection [26], [27].

The first important step in our in vivo study was to demonstrate that the pVHAVI and pVAVI systems were integrated into muscle cells by phiC31 integrase after one month, and this was proven by PCR (Fig. 5B). It is also very important to note that both systems produced VEGF at a similar level (Fig. 5A), but the physiological response was quite different between them, as follows: 1) most of the animals treated with pVHAVI showed no necrosis, whereas half of those treated with pVAVI had some degree of necrosis (Fig. 3); 2) muscle weight and force were higher in animals treated with the pVHAVI system (Fig. 4A and 4B) and 3) the degree of angiogenesis and fibrosis was better with the pVHAVI system than the pVAVI system (Fig. 6).

Even though the VEGF concentration in muscles was similar in both systems, it is important to note that this concentration was determined from the whole muscle only once, at the 4^th^ week, due to limitations of the method. Therefore, whether any variation in VEGF gene expression occurred in different parts of the muscle after transfection with either vector system during the 4 weeks is unknown. However, it is reasonable to assume that the pVHAVI system, which mainly expresses VEGF during hypoxia, acted differently than the pVAVI system, which expresses VEGF constitutively. We have no direct evidence to support this assumption, but the in vitro data and the improvement of animals treated with the pVHAVI system allow us to make this interpretation.

In this study, the viral CMV promoter was used to construct pVHAVI, which promoted hVEGF expression for three months in vitro and one month in vivo, at least. However, it is a well-known phenomenon that viral promoters, used to express mammalian genes, are frequently silenced during long-term studies. The use of muscle specific promoters like MCK (muscle creatine kinase) in construction of pVHAVI can bring better benefit to PAD patients in long-term treatment.

In conclusion, we demonstrated that a plasmid vector with an HRE sequence incorporated into it provides hypoxia-inducible VEGF expression. This vector, produced with the phiC31 integrative system, allowed for long-term VEGF gene expression, which was only activated under hypoxic conditions. For the treatment of mouse limb ischemia, the hypoxia-sensitive vector pVHAVI ameliorated the symptoms much better than the hypoxia-insensitive vector pVAVI. This last result corroborates the idea that the presence of high concentrations of VEGF in ischemic tissue is not beneficial or is less beneficial than maintaining a lower but sufficient and long-term concentration of VEGF locally.
